# Obesity is associated with more disability at presentation and after treatment in low back pain but not in neck pain: findings from the OIOC registry

**DOI:** 10.1186/s12891-016-0992-0

**Published:** 2016-03-31

**Authors:** Maria M. Wertli, Ulrike Held, Marco Campello, Shira Schecter Weiner

**Affiliations:** Horten Centre for Patient Oriented Research and Knowledge Transfer, Department of Internal Medicine, Zurich University, University Hospital Zurich, Pestalozzistrasse 24, CH-8032 Zurich, Switzerland; NYU Hospital for Joint Diseases, Occupational and Industrial Orthopaedic Center (OIOC), New York University, 63 Downing Street, New York, NY 10014 USA; Division of General Internal Medicine, Bern University Hospital, Bern University, Freiburgstrasse 8, CH-3010 Bern, Switzerland

## Abstract

**Background:**

The influence on the treatment response in patients with low back pain (LBP) and neck pain (NP) is unknown. The aim of the study was to investigate the influence of body weight in patients with low back pain (LBP) and neck pain (NP) on baseline and end of treatment disability.

**Methods:**

Cross-sectional analysis of baseline factors. Longitudinal analysis of prospectively collected patient information at an outpatient physical therapy registry (data from June 2010 to December 2012). WHO-BMI classification was used: underweight, lean, overweight, obesity class I, obesity class II and III. The influence of body weight and a predefined set of confounders was analyzed by multiple regression models.

**Results:**

In LBP, disability increased with increasing BMI [lean = reference, obesity class I Beta 5.41 (95 % CI 0.75; 10.07), obesity class II-III Beta 7.58 (95 % CI 2.13; 13.03)]. Compared to lean patients, disability after treatment improved in overweight subjects [Beta −3.90 (95 % CI −7.4; −0.41)] but not in subjects with obesity class II–III [Beta 3.43 (95 % CI −3.81; 10.68)]. There were insufficient patients in the sample with severe obesity and therefore this trend has to be confirmed. The likelihood for meaningful important change (MID) was similar in all BMI subgroups. For patients with NP, BMI was not associated with baseline disability, and did not predict end of treatment disability or the likelihood of a MID. These findings must be interpreted with caution as BMI subgroups did not meet the required sample size.

**Conclusion:**

Overweight and obesity are associated with higher levels of disability before treatment in LBP patients, but not in NP. In severely obese patients class II–III with LBP the rate of MID was lowest indicating that these patients experienced the least treatment response compared to the other groups. Further studies should address the impact of severe obesity on the prognosis of LBP. In patients with LBP, severe obesity may be an important factor to consider during the physical therapy treatment. In particular, combined treatment strategies combining weight management, cardiovascular fitness, and low back pain rehabilitation should be investigated.

## Background

The prevalence of obesity has been steadily increasing since the 1960s. Currently about one third of the adult U.S. population is obese [1]. The implications for the working population are yet to be fully realized. The lost productivity yearly due to obesity is estimated at $12 billion [2]. Obesity is associated with a high incidence of work-related musculoskeletal disorders [3]. It has been suggested that obesity is associated with more spinal pain, mainly low back pain (LBP) [4] and neck pain (NP) [5]. The cost of spinal pain for the US workforce is estimated at $20 billion annually [6]. Together, obesity and spinal pain represent two clinically and economically important public health problems that drive health care utilization. Currently the interactive effect of obesity on recovery from spinal pain is poorly understood and is needed in order to develop effective treatment interventions.

Spinal pain, primarily nonspecific NP and LBP, is a common problem. The lifetime prevalence for LBP exceeds 80 % and for NP ranges between 40 and 70 % [7, 8]. With LBP every fifth patient will eventually develop chronic pain-related disability and generate two- thirds of the direct and indirect costs [7]. While the prognosis in the early course of LBP is good, return to full function is difficult to achieve once the condition becomes chronic [7]. NP is frequently persistent [9, 10] and both NP and LBP have high rates of recurrence [7, 9, 10]. While the body of research describing the management of LBP is more robust than that for NP, in cases of nonspecific neck or LBP, it appears that similar treatment strategies may be beneficial [11, 12]. Research has shown that early screening can identify individuals at risk for chronicity [13]. Therefore, patients at risk for chronic spinal pain should be identified early for targeted treatment interventions that may improve outcomes, most specifically, preventing disabling pain and chronicity [14].

Obese individuals, compared to lean individuals, are more likely to experience NP and LBP [5, 15, 16], are expected to have a slower recovery [17–19], and are more likely to seek healthcare for LBP [19]. This suggests that not only might it cost more to treat obese patients but also that obesity might be a prognostic factor for long-term functional limitations [20] and increased health care expenditure. Therefore further exploration is needed to better understand these phenomena.

Different treatment approaches are available for managing spinal pain [7, 12]. Several prognostic and mediating factors have been identified that might hinder treatment success. For example, psychological factors, such as, for example, fear avoidance, have been shown to negatively influence treatment efficacy in patients with LBP [21, 22]. There is clear evidence that a biopsychosocial approach to care, focusing on activity and managing fears and other emotional issues related to the pain results in best outcomes including functional restoration, decreased health care utilization, and reduced direct and indirect costs [13]. Yet few studies have addressed the influence of obesity on treatment response in spinal rehabilitation and among those that have, the findings were inconsistent [23–27]. Therefore, it is unclear how obesity affects disability and treatment response in patients with nonspecific LBP or NP.

The aim of this analysis is to investigate the influence of body weight on disability in patients with spinal pain and to explore the influence of body weight on functionally-related outcomes in individuals undergoing best-evidence rehabilitation. We hypothesize that obese patients experience more disability compared to lean patients. We further hypothesize that body weight is inversely correlated with functional outcomes after treatment for nonspecific NP and LBP pain. The analysis is based on prospectively collected patient information from an outpatient rehabilitation clinic registry in New York City, New York, USA [28].

## Methods

### Design

This study is a cross-sectional analysis of baseline factors and longitudinal analysis of prospectively collected patient information during outpatient physical therapy. The description of the study inclusion process and the analysis was based on the guidelines for reporting observational studies, as described in the Strengthening The Reporting of Observational studies in Epidemiology (STROBE) statement [29].

### Eligibility criteria

All treatment records of subjects treated for LBP and NP at an urban outpatient rehabilitation facility were potentially eligible for the analysis. Included were all records of patients 18 years and older who reported weight and height and perceived disability. Excluded were records without information on body weight or height, as no body mass index (BMI) could be calculated, or those missing reports of perceived disability.

### Data collection process of the clinic registry

The data used for the current analysis were collected between June 2010 and December 2012 at the NYU Hospital for Joint Diseases Occupational and Industrial Orthopaedic Center (OIOC), New York University Langone Medical Center (NYLMC), New York, U.S.A. The OIOC is an outpatient center of excellence and provides outpatient physical therapy services for the management of musculoskeletal conditions, specializing in spine-related musculoskeletal disorders. Patients are referred by specialists, insurance companies, and primary care physicians from the tri-state area (New York, New Jersey, Connecticut), and represent a wide spectrum of the working and non-working population in this region.

As part of routine clinical practice, all OIOC patients provide a core set of information and complete a series of self-reported validated questionnaires before the start of treatment, during treatment, and at discharge [28]. This data is used by clinicians to inform treatment decisions, and assess treatment effectiveness. The collected parameters are based on the recommendations of a consensus statement by Pincus et al. [30] aimed at enhancing quality in outcome measurements. The data collected reflects the patients’ self-report of function, pain, demographic, psychosocial, and psychological factors that previous research has found to be relevant to treatment outcome [30].

The questionnaire forms are scanned or entered directly into a recording system. All data is de-identified when entered into the registry database of the International Spine Registry - Spine Tango [31, 32]. The database follows HIPAA compliance practices related to the security of data. Researchers involved in the analysis of the de-identified data were not involved in the data collection process and did not have access to any personal identifiers collected by the spine clinic.

### Treatment

All patients with NP and LBP at the OIOC receive an evidence-based approach to care including four to eight weeks of active physical therapy, and spine education and information [12, 33]. All treatment is provided by physical therapists specially trained in a multidisciplinary approach to care for spinal patients. Treatment takes place at the spine clinic approximately twice a week for one hour. Evidence suggests that patients with neck and back pain should be encouraged to remain active. Exercise prescription was based in this ideology and was customized for patients based on evidence of muscle weakness and tightness, and moderated within tolerable limits. Patients were educated that they need not be fearful of some degree of discomfort during exercise. All exercise for the neck and back comprehensively addressed both some degree of cardiovascular training combined with stretching and strengthening the spine and extremities based on individual presentation. In addition, all patients are instructed in self-care techniques that include avoidance of bed rest, activities as tolerated, ice or heat as needed, over the counter medications for symptom control, and relaxation techniques. A progressive daily home back exercise program is part of the treatment and patients are expected to exercise independently. The program is customized by the treating physical therapist based on the specific physical presentation of the patient. Treatment progress is assessed on a regular basis by the multidisciplinary team, consisting of a physician, psychologist, and physical therapists. As part of treatment, yellow flags are monitored and addressed by the PT. Yellow flags refer to psychosocial barriers to recovery. Physical therapists in this setting were trained to address yellow flags during the course of rehabilitation. In patients with persistent yellow flags despite the physical therapist intervention to address the distress and fears were referred to a health psychologist, who is a part of the interdisciplinary treatment team at the clinic.

### End of treatment assessment

The OIOC registry includes the collected forms of all patients evaluated at the spine clinic. Completing the forms was voluntarily, yet was a routine part of the intake process. Some patients were seen only for an evaluation as they chose not to start a treatment program or no approval by the insurance company was obtained. Therefore, more baseline than end of treatment assessments were available. Furthermore, some patients chose not to self-report their body weight or height and were therefore not included in this analysis. The baseline characteristics of all patients were compared to the patients included in this analysis for any indication of bias. Patients that underwent treatment and chose to complete an end of treatment questionnaire were included in this analysis. Treatment duration was calculated based on the baseline assessment date and the date of the completed end of treatment form. The treatment was stopped when 1) it was no longer necessary, 2) there was no further coverage by the insurance companies, 3) the patient chose not to pursue treatment, or 4) the patient was non- compliant with the recommendations of the therapists.

### Outcome definition

The primary outcome was patient-reported disability as assessed by the Oswestry Disability Index [ODI [34]] for patients with LBP and the neck disability index (NDI) for patients with NP [35]. Both, the ODI and the NDI are widely used, valid, and reliable questionnaires that measure disability in spine pain patients. The ODI and the NDI consist of 10 questions (each item score 0 to 5). The score ranges from 0 to 100 % [(total scored/total possible score) * 100]. For the longitudinal analysis, a meaningful clinically important difference (MCID) in the ODI or NDI was defined as a change of 30 % [36].

### Body mass index classes

Self-reported body weight and height at baseline were used to calculate the BMI, which is computed by kilograms per square meter (kg/m^2^). Patients were classified according to the WHO classification [37]: underweight (BMI < 18 kg/m^2^), lean (BMI 18- <25 kg/m^2^), overweight (BMI 25- <30 kg/m^2^), obesity class I (BMI ≥ 30 - <35 kg/m^2^), obesity class II and III (≥35 kg/m^2^).

### Confounders

The following potential confounders were defined a priori in an interdisciplinary group consensus and by consulting the relevant literature [30, 38]: Age, gender, currently not in a relationship, blue collar workers (e.g. craft or trades worker, elementary workers), low education (high school or lower education), not working (because of the medical condition, ill health, or pain) or working limited duty or alternate job because of the medical condition, receiving workers’ compensation, high baseline pain [Numeric Rating Scale (NRS), range 0–10 [39]]. High baseline pain was defined as NRS of more than 6 points, chronic pain as more than 3 months pain duration.

Fear avoidance beliefs were assessed by using the validated Fear Avoidance Beliefs Questionnaire [FABQ [40]]. The scale has been shown to be valid and reliable and consists of two sub-scales. The fear of work (FABQ-W) sub-scale ranges from 0 to 42 points with high values indicating more fear avoidance beliefs. A cut-off for FABQ-W of >25 has been proposed [41]. The fear of physical activity sub-scale (FABQ-P) ranges from 0-24 points. A FABQ-P cut-off of >15 points is suggested [41]. Both cut-points were associated with a worse prognosis in LBP [41].

### Sample size calculation

We calculated the required sample size for subgroups in a longitudinal analysis comparing the lean patients with the obese patients, assuming a 20 % difference between the groups in change in ODI/NDI over time. With a power of 80 % and a level of significance of 5 %, this results in 28 patients per group. Anticipating a drop-out rate of 15 %, this would require the inclusion of at least 33 patients per group at baseline.

### Statistical Analysis

Descriptive statistics included median and interquartile range for the continuous parameters, and percentages for the categorical outcomes. Results are presented in categories of the LBP cross-sectional, LBP longitudinal, NP cross-sectional, and NP longitudinal analyses. Results of the linear models are presented with the estimate and the corresponding 95 % confidence interval (95 % CI).

#### Description of the process used for variable selection

The above described confounders were defined a priori based on an interdisciplinary consensus and by consulting the relevant literature [30, 38].

#### Multiple imputation and variable selection including interactions

Complete data sets were available for ODI, NDI, and BMI. Because of missing values in the confounders (results section: percentage missing between 5 and 23 % in cross-sectional analysis), we multiply imputed the dataset (m = 5). The chained equation approach was used and the five datasets were stacked into one “long” data file. We used the approach proposed by Rothman et al. to consider a confounder if a variable changes the effect of the determinant on the outcome by more than 10 % [42]. Therefore, we fitted linear regression models to the outcome in this stacked dataset with BMI as the determinant, well aware of the fact that the estimated coefficient of BMI is not affected by the stacking of the dataset (the stacking only affects the standard error of the estimated coefficient). Among the set of potential confounders, we evaluated each variable added to the determinant BMI for a change in the determinant’s effect size of more than +/- 10 %. If the added variable changed the estimated BMI effect more than 10 %, it was included in the final multiple regression model as a confounding factor. For each outcome, a set of confounders was identified with this approach. Finally, the interaction of the confounders with the BMI subgroups was tested for significance.

#### Fitting of the final multiple linear regression model

When we fitted the final multiple regression models, including the determinant BMI and the set of confounders, to the outcomes or change in outcomes over time, we used Rubin’s formula for the combination of effect estimates and their standard errors from the multiply imputed datasets. We additionally evaluated each confounder for the presence of an interaction term with BMI. The interaction was deleted from the model, if it was not significantly different from zero.

Statistical analyses were conducted by using the statistical software R [43] and the R-package “mice.”

### Ethics statement

This study is based on administrative de-identified data handled in compliance with HIPPA and NYULMC regulations. According to the IRB at NYU, given the nature of the study, no approval was necessary.

## Results

**Fig. 1:**
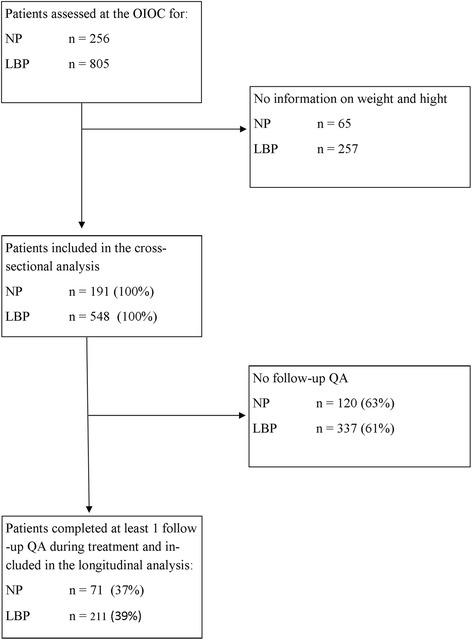
Study Flow

All patients who present to the OIOC clinic for an evaluation completed intake questionnaires on a voluntary basis. Between June 2010 and December 2012, a complete baseline data set for ODI, NDI, and BMI data was available for the cross-sectional analysis in 739 patients (548 LBP and 191 NP). Not all patients started treatment after the first evaluation. Most frequently patients chose not to start a treatment program or no approval by the insurance company was obtained. In total, 211 LBP and 71 NP patients received treatment with an end of treatment evaluation and were included in the longitudinal analysis (Fig. 1). A description of the baseline characteristics of the study population is shown in Table 1. For comparison, Appendix 1 is a description of these same baseline characteristics for all 805 LBP and 256 NP patients. As there was great variability in the number of subjects per BMI class, a summary of subjects per group is shown in Table 2. In LBP the rate of end of treatment assessment was 45 % in underweight patients, 42 % in lean patients, 40 % in overweight patients, 33 % in obese class I patients, and 25 % in obese class II–III patients. In NP the rate of end of treatment assessment was 67 % in underweight patients, 35 % in lean patients, 39 % in overweight patients, 32 % in obese class I, and 38 % in obese class II–III. A BMI-related effect might have not been detected (Type II error) for those BMI subgroups that did not meet sample size requirements for subgroup analysis. Treatment duration was similar for patients with LBP and patients with NP (median 44 and 46 days respectively).

**Table 1:** Baseline Characteristics of the patient population with reported weight and height

	LBP cross-sectional	LBP longitudinal	Neck cross-sectional	Neck longitudinal
General Characteristics				
Patients	548	211	191	71
Age: median (IQR)	49 (37; 61)	51 (38; 61)	46 (37; 56)	47 (38; 59)
No age reported: *n* (%)	28 (5)	2 (1)	14 (7)	0 (0)
Gender female: *n* (%)	288 (53)	113 (54)	114 (60)	47 (66)
Missing gender information: *n* (%)	25 (4)	2 (1)	13 (7)	0 (0)
BMI: median (IQR)	26.6 (23.1; 30.2)	26 (22.8; 29.1)	24.8 (22.4; 28.3)	24.7 (22.5; 28.1)
Married or in a relationship: *n* (%)	229 (42)	90 (43)	81 (47)	34 (48)
No relationship, divorced: *n* (%)	275 (50)	110 (52)	90 (47)	34 (48)
Not reported: *n* (%)	44 (8)	11 (5)	20 (11)	3 (4)
Low education (high school): *n* (%)	126 (23)	45 (21)	28 (15)	13 (18)
Higher education: *n* (%)	361 (66)	150 (71)	138 (71)	52 (73)
No education reported: *n* (%)	61 (11)	16 (8)	25 (16)	6 (9)
Insurance Type				
Medicare/Medicaid: *n* (%)	162 (29)	68 (32)	29 (15)	11 (15)
No fault: *n* (%)	4 (1)	3 (1)	10 (5)	5 (7)
Workers compensation: *n* (%)	33 (6)	14 (7)	11 (6)	6 (8)
Other (private, others): *n* (%)	322 (59)	124 (59)	128 (67)	49 (69)
None: *n* (%)	1 (0.001)	0 (0)	0 (0)	0 (0)
Not reported: *n* (%)	26 (5)	2 (1)	13 (7)	0 (0)
Occupation				
White collar worker: *n* (%)	159 (29)	62 (29)	71 (37)	24 (34)
Blue collar worker: *n* (%)	85 (16)	29 (14)	24 (13)	10 (14)
Other: *n* (%)	0 (0)	0 (0)	0 (0)	0 (0)
Not reported: *n* (%)	58 (10)	15 (7)	24 (13)	6 (8)
Work Status				
Working full time/part time: *n* (%)	273 (50)	106 (50)	118 (62)	46 (64)
Retired: *n* (%)	55 (10)	24 (4)	12 (6)	6 (8)
Mainly not working during the last 12 months: *n* (%)	373 (68)	152 (28)	57 (30)	22 (31)
Currently not working: *n* (%)	231 (42)	89 (42)	103 (54)	37 (52)
Not working because of ill health or pain: *n* (%)	95 (17)	40 (19)	20 (10)	7 (10
Not working because of main complaint				
Job-related injury: *n* (%)	28 (5)	13 (6)	10 (5)	1 (1)
Workers compensation case to the main complaint: *n* (%)	22 (4)	11 (5)	7 (4)	6 (8)
Limited duty: *n* (%)	18 (3)	7 (3)	4 (2)	1 (1)
Pain-related				
Acute: to 6 weeks: *n* (%)	39 (8)	17 (8)	17 (9)	4 (6)
Subacute: 6 to 12 weeks: *n* (%)	79 (14)	34 (16)	30 (16)	17 (24)
Chronic: more than 12 weeks: *n* (%)	363 (66)	137 (65)	119 (62)	46 (65)
No pain duration reported: *n* (%)	67 (12)	23 (11)	25 (13)	4 (6)
Sciatica/neck and arm pain: *n* (%)	44 (8)	27 (13)	4 (2)	0 (0)
Time under therapy: median (IQR) days		44 (32; 67)		46 (35; 63)
Self-reported measures				
Baseline ODI/NDI: median, IQR	30 (18; 47)	28 (18; 45)	15 (9; 22)	13.3 (10; 20)
End of treatment ODI/NDI: median, IQR	–	20 (10; 36)	–	10 (5; 15)
No end of treatment ODI/NDI	–	13	–	2
Baseline NRS: median, IQR	6 (4; 8)	7 (4; 8)	6 (4; 7)	6 (3; 7)
No baseline NRS	50	17	21	3
End of treatment NRS: median, IQR	–	4.5 (3; 7)	–	4 (2; 5)
No end of treatment NRS	–	165	–	50
Baseline FABQ-W: median, IQR	9 (0; 21)	7 (0; 18)	11 (3; 21)	13 (1; 22)-
High FABQ-W (>25): *n* (%)	80 (15)	27 (13)	30 (16)	15 (21)
No baseline FABQ-W	57 (10)	26 (12)	24 (13)	3 (4)
End of treatment FABQ-W: median, IQR	–	6 (0; 17)	–	10 (0; 16)
No end of treatment FABQ-W	–		–	–
Baseline FABQ-P: median, IQR	15 (12; 19)	15 (11; 18)	15 (11; 19)	15 (9; 19)
High FABQ-P (>15): *n* (%)	231 (42)	92 (44)	75 (39)	18 (25)
No baseline FABQ-P	40	12	20 (10)	2 (2.8)
End of treatment FABQ-P: median, IQR	–	12 (6.75, 16.3)	–	11 (3; 21)
No end of treatment FABQ-P	–	43	–	15 (4.7)

*n* number of subjects; *IQR* interquartile range; *ODI* Oswestry Disability Index (range 0–100), *NDI* Neck Disability Index (range 0–100); *FABQ* fear avoidance questionnaire; *FABQ-W* work sub-scale (range 0–42); *FABQ-P* physical activity subscale (range 0–24); *BMI* body mass index

**Table 2:** Distribution of the patients according to the BMI subgroups

	LBP cross-sectional	LBP longitudinal	NP cross-sectional	NP longitudinal
Patients: *n*	548	211	191	71
Underweight (BMI < 18 kg/m^2^): *n* (%)	11 (2)^a^	5 (2)^a^	6 (3)^a^	4 (6)^a^
Lean (BMI 18- < 25 kg/m^2^): *n* (%)	198 (36)	83 (39)	94 (49)	33 (46)
Overweight (BMI 25- <30 kg/m^2^): *n* (%)	194 (35)	79 (37)	61 (32)	24 (34)^a^
Obesity class I (BMI 30- < 35 kg/m^2^): *n* (%)	94 (18)	31 (15)^a^	22 (12)^a^	7 (10)^a^
Obesity class II–III (BMI ≥ 35 kg/m^2^): *n* (%)	51 (9)	13 (6)^a^	8 (4)^a^	3 (4)^a^

^a^groups that did not meet the calculated sample size for sub-group analysis of 33 patients (power of 80 %, level of significance of 5 %)

### Cross-sectional analysis results

In the LBP group, at baseline, obese patients expressed more disability [obesity class I Beta 5.41 (95 % CI 0.75; 10.07), obesity class II–III Beta 7.58 (95 % CI 2.13; 13.03)] compared to lean patients (Table 2). Low education was associated with more disability (Beta 11.20, 95 % CI 5.45; 16.94). The interaction between obesity class II–III and low education reduced this effect [Beta −12.33 (95 % CI −22.80; −1.87)]. No other interactions between the predefined confounders and the BMI classes were found for the LBP group. In patients with NP, we found no significant influence of BMI categories on baseline disability (Table 3). Baseline pain (Beta 5.84 95 % CI 3.03; 8.66) and high fear avoidance beliefs of physical activities (FABQ-P, Beta 4.53 95 % CI 2.07; 6.98) were associated with more disability. Furthermore, no interaction between other confounders and BMI were found.

**Table 3:** Results Cross-sectional Analysis for Disability (ODI or NDI)

	BMI category	Beta (95 % CI)	*p*-value
LBP^a^	Lean (reference)	–	–
	Underweight	3.02 (−8.06; 14.10)	0.59
	Overweight	1.17 (−2.17; 4.52)	0.49
	Obesity class I	**5.41 (0.75; 10.07)**	**0.02**
	Obesity class II–III	**7.58 (2.13; 13.03)**	**0.01**
	Low education	**11.20 (5.45; 16.94)**	**0.00**
	Blue collar jobs	1.23 (−2.56; 5.01)	0.53
	Not working	3.06 (−0.62; 6.73)	0.10
	Work-related RF	**7.82 (0.33; 15.32)**	**0.04**
	High pain baseline	**15.59 (12.86; 18.31)**	**0.00**
	FABQ-W high	5.30 (−0.13; 10.73)	0.06
	FABQ-W N/A	**4.98 (0.31; 9.66)**	**0.04**
	Underweight x Low education	−11.99 (−36.47; 12.50)	0.33
	Overweight x Low education	−4.48 (−11.94; 2.99)	0.24
	Obesity class I x Low education	−6.12 (−14.48; 2.25)	0.15
	Obesity class II–II x Low education	**−12.33 (−22.80;−1.87)**	**0.02**
NP^b^	Lean (reference)		
	Underweight	4.57 (−1.70; 10.83)	0.15
	Overweight	−1.11 (−3.61; 1.40)	0.38
	Obesity class I	−0.32 (−3.90; 3.26)	0.86
	Obesity class II–III	−0.95 (−6.90; 5.00)	0.75
	Age	−0.04 (−0.13; 0.06)	0.43
	Male gender	−1.32 (−3.82; 1.19)	0.30
	No relationship	2.19 (−0.17; 4.55)	0.07
	Blue collar jobs	1.21 (−2.65; 5.07)	0.53
	Not working	2.21 (−0.78; 5.20)	0.14
	Work-related RF	3.37 (−1.12, 7.85)	0.14
	High baseline pain	**5.84 (3.03; 8.66)**	**<0.001**
	High FABQ-P	**4.53 (2.07; 6.98)**	**<0.001**
	High FABQ-W	3.45 (−0.26; 7.15)	0.07
	FABQ-W N/A	−1.23 (−5.35; 2.89)	0.55

Pre-defined confounders were: low education (high school or less), blue collar jobs includes crafts or trade worker, agricultural or elementary worker, high FABQ-W, FABQ-W N/A = not working, high pain (NRS > 6), age, gender, not in a relationship

High pain (NRS > 6), FABQ, fear avoidance questionnaire, fear avoidance work sub-scale (FABQ-W) high > 25; fear avoidance beliefs physical sub-scale (FABQ-P) high >15

Work related risk factor: working full time at alternate or limited duty job because of a medical condition; not working because of pain or ill health; looked but can’t find a job; not working because of the main complaint in a job-related injury; pending workers compensation case

^a^Confounder included in the final model when they changed the effect between BMI and ODI/NDI by at least 10 %. Interactions with BMI were included if they were significantly different from zero (p < 0.05)

^b^Confounder included in the final model when they changed the effect between BMI and ODI/NDI by at least 10 %. In NP no interactions of confounders with BMI were found

### Results longitudinal analysis

**Fig. 2:**
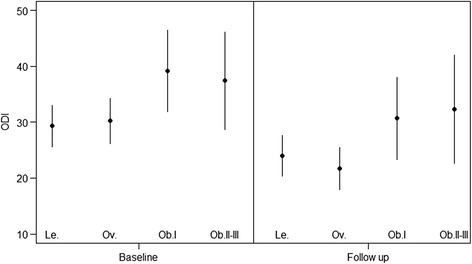
Distribution of ODI at baseline and end of treatment. Raw ODI data: ODI mean and 95 % Cl; Le, lean; Ov, overweight; Ob.I, obesity class I; Ob.II–III, obesity class II–III

Among patients with LBP, the subgroup “overweight” was associated with a greater decrease in ODI scores (decreasing disability) at the end of treatment assessment [Beta −3.90 (95 % CI −7.4; −0.41)] compared to lean patients (Table 4). Obesity class II–III was associated with less reduction in disability at the end of treatment assessment; however, the confidence interval was wide and not statistically significant [Beta 3.43 (95 % CI −3.81; 10.68)]. Figure 2 depicts the mean and 95 % CI intervals for ODI at baseline and the end of treatment assessment in all BMI subgroups. The results for the obesity class II–III group need to be interpreted cautiously due to insufficient subjects available for this sub-group analysis. None of the predefined confounders showed a statistically significant interaction. The likelihood for a clinical meaningful change in ODI from baseline to the end of treatment assessment was not different between the BMI categories (Table 5).

**Table 4:** BMI category as prognostic factor for disability (ODI/NDI) at the end of treatment

	BMI category	Beta (95 % CI)	*p*-value
LBP^a^	Lean (reference)	–	
	Underweight	−4.92 (−18.1; 8.27)	0.45
	Overweight	**−3.90 (−7.4;−0.41)**	**0.03**
	Obesity class I	−0.83 (−6.1; 4.46)	0.76
	Obesity class II–III	3.43 (−3.81; 10.68)	0.35
	ODI baseline	**0.59 (0.49; 0.69)**	**<0.001**
	FAB-P high	**7.76 (4.39; 11.13)**	**<0.001**
	FAB-W high	−0.82 (−7.1; 5.4)	0.79
	FAB-W high NA	2.42 (−2.69)7.53)	0.35
	No relationship	**3.03 (−0.43; 6.49)**	**0.09**
	Work-related RF	3.03 (−2.25; 8.32)	0.25
	Chronic pain	4.49 (0.34; 8.64)	0.03
	Treatment duration	0.04 (−0.02; 0.09)	0.16
NP^a^	Lean (reference)	–	
	Underweight	−0.23 (−6.58; 6.12)	0.94
	Overweight	0.91 (−2.23; 4.05)	0.57
	Obesity class I	−0.46 (−5.39; 4.48)	0.85
	Obesity class II–III	−4.65 (−11.98; 2.69)	0.21
	NDI baseline	**0.61 (0.43; 0.78)**	**<0.001**
	Work-related RF	4.07 (−0.35; 8.48)	0.07
	Treatment duration	0.03 (−0.02; 0.08)	0.25

Positive Beta coefficient indicates less decrease in ODI/NDI; negative Beta coefficient indicates more decrease in ODI/NDI than in the reference group (lean patients)

^a^No significant interactions between BMI and other potential prognostic factors were found for low back and neck pain patients

All confounders included in the final model changed the influence of BMI on the outcome ODI/NDI at least 10 % in univariate comparison

**Table 5:** Results longitudinal analysis for 30 % change as MID in ODI or NDI

	BMI category	% with MID^a^	OR (95 % CI)	*p*-value
LBP^b^	Lean (reference)	36.3	–	–
	Underweight	33.3	1.19 (0.68; 2.09)	0.63
	Overweight	48.0	1.15 (0.99; 1.34)	0.35
	Obesity class I	46.4	1.14 (0.92; 1.40)	0.25
	Obesity class II–III	33.3	0.98 (0.73; 1.33)	0.91
NP	Lean (reference)	41.9		
	Underweight	50	1.12 (0.58–2.19)	0.73
	Overweight	41.7	1.00 (0.72–1.39)	0.98
	Obesity class I	57.1	1.03 (0.64–1.64)	0.91
	Obesity class II–III	66.7	1.30 (0.69–2.44)	0.41

Results adjusted for the following confounders with at least 10 % change in the effect between BMI on ODI/NDI

^a^raw rate of MID

^b^not in a relationship, blue collar work, not working, work-related risk factor, chronic LBP, high pain (NRS >6), high FABQ-P

**Fig. 3:**
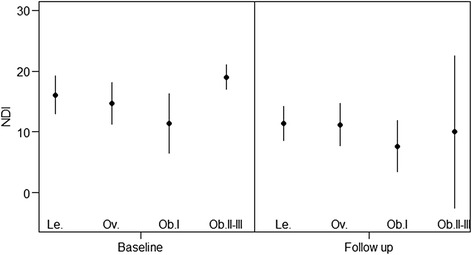
Distribution of NDI at baseline and end of treatment. Raw NDI data: NDI mean and 95 % Cl; Le, lean; Ov, overweight; Ob.I, obesity class I; Ob.II–III, obesity class II–III

In patients with NP, none of the tested confounders predicted the end of treatment NDI (Tables 4 and 5). NDI values decreased in all groups similarly (Fig. 3). BMI subgroups were compared to lean patients and predicted no clinically meaningful change in NDI. Figure 3 illustrates the distribution of NDI with mean and 95 % CI for the BMI subgroups. The findings of the longitudinal analysis have to be interpreted with caution because in most sub-groups the sample size requirements were not met.

## Discussion

The analysis of a registry of patients treated for LBP or NP showed that in LBP, obesity affects clinically important factors. Patients with LBP expressed more disability with increasing BMI at baseline when compared to lean subjects. During a course of treatment, LBP disability improved more in overweight subjects when compared to lean patients. In comparison we found in severely obese patients (obesity class II–III) less improvement in disability. While this finding in the severely obese patients was not statistically different compared to lean patients, the wide confidence intervals indicate that heterogeneity in the patient population was present suggesting that further studies are needed to explain this finding. Independent prognostic factors for the end of treatment ODI were baseline ODI and high baseline fear avoidance of physical activity (FABQ-P).

In NP patients we found no influence of BMI on baseline disability. Baseline disability was associated with higher pain scores and higher fear of physical activity beliefs scores. The only significant prognostic factor for disability at the end of treatment was the baseline disability. Independent prognostic factors for the end of treatment disability were baseline NDI, high baseline pain, and high fear avoidance beliefs of physical activity. However, the findings in the NP population need to be interpreted with caution as the sample size requirements for sub-group analyses were not met. Therefore, we must consider that we might have missed a relevant influence of BMI on NP-related disability.

### Results in light of the literature

Whether or not obese patients respond differently to non-pharmacological treatment approaches for spinal pain is controversial [25–27, 44]. While one cohort study found that obese patients responded similarly to treatment compared to their lean counterparts [44], other studies found that obesity was associated with a failure to improve [26, 27, 45]. Furthermore, retrospective studies suggest that obese patients express higher levels of fear avoidance than their lean counterparts and therefore are at risk for worse treatment response [25] and respond poorer to cognitive behavioral therapy, a treatment aimed at addressing fear of movement [24]. Our findings support that a higher BMI is associated with more disability at baseline in patients with LBP. A previously observed interaction between fear avoidance beliefs and obesity [25] was not found in this study. Additionally, while overweight subjects with LBP reported higher baseline ODI than lean subjects, the end of treatment ODI was lower (on average −3.90 points, 95 % CI −7.4; −0.41) and they reported the highest rate of MID (48 %). The OR for MID was 1.15 and the 95 % CI crossed 1 and therefore the finding was not statistically significant (95 % CI 0.99; 1.34). During treatment, severely obese patients (class II–III) experienced less decrease in disability than the other groups (+3.43 points (95 % CI −3.81; 10.68) and had the lowest MID rate (33 %) indicating that these patients experienced a worse treatment response compared to the other groups. Again, the OR for MID was not statistically significant (OR 1.30 (95 % CI 0.69–2.44). In addition, the rate of follow-up assessment was 25 % in the severely obese patients compared to 33 % in obese class I patients and 40 % in overweight patients which may indicate a higher rate of unfinished treatments in severely obese patients. It may be hypothesized that overweight patients are moderately deconditioned and respond well to physical therapy treatment. Severely obese patients may have, in addition to LBP, other problems including gait insecurity and sarcopenia which may require other treatment strategies than those included in their LBP regimen [46]. This finding should be further evaluated in follow-up studies, as it suggests that the amount of excess weight modifies the treatment response. Our patient population included insufficient patients with severe obesity to draw conclusions, and therefore our findings have to be confirmed in studies specifically addressing this patient population. The long-term effects of our findings are unknown and to the best of our knowledge no study has been conducted with sufficient numbers of obese patients to address the question of long-term outcome.

Significant weight reduction has been shown to improve physical and mental outcome for those with LBP [47]. It has been shown that improved fitness slowed the decline in mobility in overweight adults with type 2 diabetes [48]. It may be that overweight patients are able to perform recommended spine exercises when compared to obese and severely obese subjects. Previous research has shown an association between body fat and the intensity and disability from LBP [49], and that weight reduction after bariatric surgery improved joint pain, gait, and perceived mobility [46]. Severely obese patients with LBP might start at a state of deconditioning where an exercise protocol is difficult to follow and the typical treatment duration may be insufficient for a clinically important or sustainable effect. Our findings indicate that further research is needed to study the effect of body weight on the ability of obese patients with LBP to participate in exercises, to ascertain the effectiveness of current treatment strategies and optimal treatment duration. In addition, the economic impact of a treatment approach addressing both body weight and LBP may be substantial and yield better long-term outcomes not only of LBP but also for other comorbidities associated with obesity such as cardiovascular and metabolic disorders.

Only a few studies have addressed the impact of prognostic factors on NP patients [9, 10, 50], and to the best of our knowledge, no study has investigated the influence of body weight on the treatment response in patients with NP. Our findings concur with epidemiological studies that found no influence of body weight on disability in NP patients [51]. However, due to the small patient samples in the BMI subgroups, our findings have to be interpreted with caution and further research is needed to clarify, in particular, the role of severe obesity on NP.

### Strength and limitations

The strength of this study is that all patients treated at an urban physical therapy clinic with LBP or NP were included in the analysis. Therefore, this study is based on real patients in a busy spine pain clinic. Great care was taken to address the potential influence of patient heterogeneity by systematically considering evidence-supported prognostic factors [30]. Subgroup analyses may lead to both overestimation and underestimation of an effect. To the best of our knowledge, this is the first study on the influence of body weight on disability in spinal pain that used sample size calculation for subgroup analyses to assess the validity of the results. Further, all subgroups and prognostic factors were defined a priori, tests of interaction were performed, and subgroup-specific estimates with the corresponding confidence intervals are provided to illustrate the differences.

The following describes three study limitations to address in follow-up studies. First, the sample size of patients with NP or LBP followed-up during treatment was small in the high BMI subgroups (obesity class II–III). Therefore, wide confidence intervals were observed and the small sample size might explain the lack of statistically significant findings. Further, the number of patients who completed the treatment was very low in some categories and these results need further confirmation in larger samples. Second, the data was used from a clinical registry based on self-reported questionnaires completed on a voluntary basis. The reported weight and height was not confirmed and no further analysis of the body fat distribution was available. Overweight individuals may report lower weight and therefore, the BMI in these subjects may be underestimated. Therefore, the proportion of obese patients may have been higher in our sample. While we cannot ignore that obese patients may be more likely to withhold reporting their body weight, we found no systematic difference in the baseline characteristics of all patients included in the registry and the patients included in this analysis. Third, the sample size calculation for the subgroup analysis, based on preset criteria, required a sample size of 33 patients per group for a longitudinal analysis comparing the lean patients with the obese patients. There were insufficient patients with NP available to rule out a type 2 error and therefore our findings for the neck pain population need to be interpreted with caution.

### Implications for research

Further research should address the influence of severe obesity on the treatment response in patients with LBP. Our study showed that obese patients with LBP feel more disabled. Experimental research suggests that obesity is associated with altered muscle protein synthesis [52]. Further, it has been suggested, that the presence of obesity-related comorbidities (e.g. diabetes, hypertension) are associated with more morbidity and mortality [53, 54]. The influence of obesity on response to treatment for LBP with and without the presence of comorbidities should be further investigated. Weight loss after bariatric surgery has been shown to be associated with less LBP, NP, and other joint pain and may therefore need to be considered as an added therapeutic strategy in obese patients with LBP [55]. We found no indication that obesity was associated with worse treatment outcome in NP patients. However, the patient sample available for this analysis was small and this should be addressed in larger studies.

### Implications for clinical practice

While our findings have some limitations, they provide some insight into a patient population treated at a busy back pain clinic. Our findings indicate that obese patients with LBP express more disability at baseline and we found some indication that they may not recover as well as overweight and lean subjects. Treatment interventions might therefore incorporate approaches addressing body weight or use alternative approaches to exercise routines. It has been shown that improved fitness slowed the decline in mobility in overweight adults with type 2 diabetes [48]. Our findings suggest that severe obesity might require specific guidance and that severely obese patients might experience more disability associated with LBP when compared to lean and overweight patients. When not considered, this might lead to a negative feedback for the patient and lead to early termination of physical therapy in these patients and worse outcomes, including chronic, disabling pain.

## Conclusion

Overweight and obesity are associated with higher levels of disability before treatment in LBP patients, but not in NP. In obese class II–III patients with LBP, the rate of MID was lowest, indicating that these patients experienced worse treatment response compared to the other groups. Further studies should address the impact of severe obesity on LBP recovery. In particular, treatment strategies combining weight management, cardiovascular fitness, physical limitations and low back pain rehabilitation should be investigated.

### Availability of supporting data

The data set on which the conclusions of the paper rely on may be made available upon request.

## Appendix

**Table 6:** Baseline characteristics of all patients in the OIOC registry

	LBP cross-sectional	LBP longitudinal	NP cross-sectional	NP longitudinal
General Characteristics				
Patients	805	305	256	91
Age: median (IQR)	52 (39; 63)	52 (41; 62)	50 (38; 59)	51 (39; 62)
No age reported	55	9	26	2
Gender female: *n* (%)	429 (53)	168 (55)	144 (56)	56 (62)
No gender	52	9	25	2
BMI: median (IQR)	26.6 (23.1; 30.2)	26.1 (22.8; 29.1)	24.8 (22.4; 28.3)	24.7 (22.5; 28.1)
No BMI: *n* (%)	257 (32)	94 (31)	65 (25)	20 (23)
Married or in a relationship: *n* (%)	321 (40)	130 (43)	99 (39)	43 (47)
No relationship: *n* (%)	397 (49)	150 (49)	122 (48)	42 (46)
Not reported: *n* (%)	87 (11)	25 (8)	35 (14)	6 (7)
Low education (no high school diploma): *n* (%)	75 (9)	19 (6)	10 (4)	2 (2)
High school diploma and higher education: *n* (%)	609 (76)	248 (81)	196 (74)	74 (81)
No education reported: *n* (%)	121 (15)	38 (12)	50 (20)	15 (16)
Insurance Type				
Medicare/Medicaid: *n* (%)	275 (34)	104 (34)	51 (19)	19 (21)
No fault: *n* (%)	5 (0.01)	3 (0.01)	11 (4)	5 (5)
Workers compensation: *n* (%)	45 (6)	22 (7)	14 (5)	7 (8)
Other (private, others): *n* (%)	425 (53)	166 (54)	155 (61)	58 (64)
Not reported: *n* (%)	54 (7)	10 (3)	25 (10)	2 (2)
Occupation				
White collar worker: *n* (%)	212 (26)	82 (27)	88 (34)	29 (32)
Blue collar worker: *n* (%)	111 (13)	39 (13)	27 (11)	11 (12)
Not reported: *n* (%)	109 (13)	32 (11)	40 (16)	9 (10)
Work Status				
Retired: *n* (%)	109 (14)	48 (16)	24 (9)	11 (12)
Not working: *n* (%)	331 (41)	129 (42)	71 (28)	29 (32)
Not working because of ill health or pain: *n* (%)	128 (16)	52 (17)	24 (9)	9 (10)
Workers compensation case to the main complaint: *n* (%)	26 (3)	14 (5)	8 (3)	6 (7)
Working full time/part time	367 (46)	146 (48)	144 (56)	55 (60)
Limited duty	21 (3)	7 (2)	6 (2)	2 (2)
Self-reported measures				
Baseline ODI/NDI: median, IQR	30 (18; 46)	28.9 (18; 44)	16 (9; 24)	14.4 (9.5; 21.6)
End of treatment ODI/NDI: median, IQR	–	22 (10; 35.6)	–	11 (4.4; 16.1)
No end of treatment ODI/NDI	–	18	–	3
Baseline NRS: median, IQR	6 (4; 8)	6 (4; 8)	6 (3; 8)	6 (3; 8)
No baseline NRS	86	24	30	6
End of treatment NRS: median, IQR	–	4 (3; 6)	–	4 (2; 5)
No end of treatment NRS	–	239	–	68
Baseline FABQ-W: median, IQR	9 (0; 21)	8 (0; 19)	12 (3; 21)	11 (0; 21)
No baseline FABQ-W	153	53	46	10
End of treatment FABQ-W: median, IQR	–	6 (0; 17)	–	9 (0; 16.3)
No end of treatment FABQ-W	–	82	–	27
Baseline FABQ-P: median, IQR	15 (11; 19)	15 (11; 18)	14 (10; 19)	14.5 (9; 19)
No baseline FABQ-P	64	20	30	3
End of treatment FABQ-P: median, IQR	–	12 (6; 17)	–	11 (7; 15.8)
No end of treatment FABQ-P	–	63	–	21
